# Interplay between neural-cadherin and vascular endothelial-cadherin in breast cancer progression

**DOI:** 10.1186/bcr3367

**Published:** 2012-12-06

**Authors:** Maryam Rezaei, Katrin Friedrich, Ben Wielockx, Aleksandar Kuzmanov, Antje Kettelhake, Myriam Labelle, Hans Schnittler, Gustavo Baretton, Georg Breier

**Affiliations:** 1 Department of Pathology, University of Dresden, Fetscherstrasse 74, D-01307 Dresden, Germany; 2 Emmy-Noether Research Group, Department of Pathology, University of Dresden, Fetscherstrasse 74, D-01307 Dresden, Germany; 3 Koch Institute for Integrative Cancer Research, 500 Main Street, Massachusetts Institute of Technology, Cambridge, MA 02139, USA; 4 Department of Anatomy, University of Münster, Vesaliusweg 2-4, D-48149 Münster, Germany; 5 Present address: Department of Biomedicine, University of Basel, Mattenstrasse 28, CH-4058 Basel, Switzerland

## Abstract

**Introduction:**

Deregulation of cadherin expression, in particular the loss of epithelial (E)-cadherin and gain of neural (N)-cadherin, has been implicated in carcinoma progression. We previously showed that endothelial cell-specific vascular endothelial (VE)-cadherin can be expressed aberrantly on tumor cells both in human breast cancer and in experimental mouse mammary carcinoma. Functional analyses revealed that VE-cadherin promotes tumor cell proliferation and invasion by stimulating transforming growth factor (TGF)-β signaling. Here, we investigate the functional interplay between N-cadherin and VE-cadherin in breast cancer.

**Methods:**

The expression of N-cadherin and VE-cadherin was evaluated by immunohistochemistry in a tissue microarray with 84 invasive human breast carcinomas. VE-cadherin and N-cadherin expression in mouse mammary carcinoma cells was manipulated by RNA interference or overexpression, and cells were then analyzed by immunofluorescence, reverse transcriptase-polymerase chain reaction, and western blot. Experimental tumors were generated by transplantation of the modified mouse mammary carcinoma cells into immunocompetent mice. Tumor growth was monitored, and tumor tissue was subjected to histological analysis.

**Results:**

VE-cadherin and N-cadherin were largely co-expressed in invasive human breast cancers. Silencing of N-cadherin in mouse mammary carcinoma cells led to decreased VE-cadherin expression and induced changes indicative of mesenchymal-epithelial transition, as indicated by re-induction of E-cadherin, localization of β-catenin at the cell membrane, decreased expression of vimentin and SIP1, and gain of epithelial morphology. Suppression of N-cadherin expression also inhibited tumor growth *in vivo*, even when VE-cadherin expression was forced.

**Conclusions:**

Our results highlight the critical role of N-cadherin in breast cancer progression and show that N-cadherin is involved in maintaining the malignant tumor cell phenotype. The presence of N-cadherin prevents the re-expression of E-cadherin and localization of β-catenin at the plasma membrane of mesenchymal mammary carcinoma cells. N-cadherin is also required to maintain the expression of VE-cadherin in malignant tumor cells but not vice versa. Thus, N-cadherin acts in concert with VE-cadherin to promote tumor growth.

## Introduction

Cadherins are a family of transmembrane proteins that, together with their associated intracellular catenins, have important functions in cell-cell adhesion. Different cell types express different members of the cadherin family. Epithelial (E)-cadherin is a key component of adherens junctions in epithelial cells and functions as a suppressor of tumor growth and invasion. Perturbation of its function leads to an invasive phenotype in many tumors [1-3]. Neural (N)-cadherin is expressed in neural tissues and fibroblasts, where it mediates a less stable and more dynamic form of cell-cell adhesion [1-4]. Vascular endothelial (VE)-cadherin is the primary component of endothelial cell adherens junctions and has an important function in regulating vascular permeability and angiogenesis [5]. Because of the important role played by cadherins in cell recognition, adhesion, and signaling, modulation of their function and expression has significant implications for the progression of tumors [1,6-10]. For instance, a switch from E-cadherin to N-cadherin expression contributes to increased tumor cell migration, invasion and metastasis [8-10]. Aberrant expression of VE-cadherin was first detected in aggressive melanoma cells and in some cases of sarcoma [11-13]. A recent study from our group has revealed that VE-cadherin is expressed aberrantly in a subset of tumor cells in human breast cancer [7]. In a mouse mammary carcinoma model, VE-cadherin expression was induced in cancer cells that had undergone epithelial-mesenchymal transition (EMT). Functional experiments showed that VE-cadherin promotes malignant tumor cell proliferation and invasion by enhancing the protumorigenic transforming growth factor-beta (TGF-β) pathway. However, the functional interaction between VE-cadherin and N-cadherin during tumor progression is poorly characterized to date.

EMT was first described by Elizabeth Hay in the 1980s as a central process in early embryonic morphogenesis [14]. The initial step of EMT includes the loss of epithelial markers such as E-cadherin via its transcriptional repression and the gain of mesenchymal markers such as vimentin. As a consequence, the cadherin-binding partner β-catenin can dissociate from the E-cadherin complex at the plasma membrane and translocate to the nucleus where it participates in EMT signaling and activates genes involved in tumor progression [15]. Epithelial cells then lose their typical baso-apical polarization as cell-cell junctions disassemble. Additionally, the cytoskeleton undergoes dynamic cortical actin remodeling and gains the front-rear polarization that facilitates cell movement [16]. Finally, cell-matrix adhesion changes as proteolytic enzymes such as matrix metalloproteases are activated [17,18]. The transition from an epithelial to mesenchymal phenotype is reversible; for example, several rounds of EMT and mesenchymal-epithelial transition (MET) occur during development as cells differentiate and the complex three dimensional structure of internal organs forms [19]. There is increasing evidence that EMT also facilitates the dissemination of tumor cells to form distant metastasis [20]. Various publications have described a switch between the epithelial and mesenchymal phenotypes through EMT and MET in models of colorectal [21], bladder [22], ovarian [23] and breast cancer [24]. These findings indicate that the phenotypic conversion of tumor cells in the metastatic cascade is multifaceted, with EMT being critical for the initial transformation from benign to invasive carcinoma and the spreading of tumor cells, but MET occurring at the site of metastatic colonization [6].

The mouse mammary carcinoma model that we have previously used to study the expression of cadherins [7] utilizes tumor cell lines that represent different stages of tumor progression: Ep5 cells are tumorigenic mammary epithelial cells transformed by the v-Ha-Ras oncogene, whereas Ep5ExTu cells, isolated from Ep5 cell tumors grown in mice, have undergone EMT *in vivo *and present a mesenchymal, invasive and angiogenic phenotype [25,26]. We observed that VE-cadherin expression is induced in these murine breast cancer cells during (TGF-β-mediated) EMT [7]. On the other hand, E-cadherin expression was downregulated, and N-cadherin levels remained unchanged. Silencing VE-cadherin expression inhibited tumor cell proliferation and invasion *in vitro*, and experimental tumor growth in mice. However, the role of N-cadherin and its potential interaction with VE-cadherin in this model is unclear. Here, we investigate the influence of N-cadherin on EMT and tumor progression in Ep5ExTu cells. Silencing N-cadherin significantly decreased VE-cadherin expression and stimulated Ep5ExTu cells to re-express E-cadherin at the cell surface. This promoted localization of β-catenin at the plasma membrane and induced the cells to undergo MET. Efficient silencing of N-cadherin expression in Ep5ExTu cells consistently inhibited tumor growth, and complete tumor regression was even seen in some cases. Taken together, these results reveal a novel interplay between classical cadherins in breast cancer progression.

## Materials and methods

### Cell culture

Ep5 and Ep5ExTu cells were cultured as described [26] in Dulbecco's modified Eagle's medium (DMEM-F12; Lonza, Basel, Switzerland) supplemented with 15% fetal calf serum (FCS). 293T cells were kept in DMEM Glutamax (Gibco, Darmstadt, Germany) supplemented with 10% FCS.

### Generation of VE-cadherin or N-cadherin-silenced Ep5ExTu cells

Oligonucleotides (Eurogentec, Seraing, Belgium) encoding small interfering RNA (siRNA) molecules specific for mouse VE-cadherin (5'-GUCUCUGAGU ACUUCCUUA-3') or N-cadherin (5'-GGAUGUGCAG GAAGGACAG-3' and 5'-UGUCAAUGGG GUUCUCCAC-3') were designed and verified to be specific for each cadherin by a Blast search (National Center for Biotechnology Information, Bethesda, MD, USA) against the mouse genome. A scrambled oligonucleotide sequence without significant homology to murine sequences (5'-AGUCGCUUAG AAACGAGAA-3') was used as a control. These oligonucleotides were then cloned into the lentiviral vector, pLVTHM, according to the guidelines provided by Tronolab (Laboratory of Virology and Genetics, École Polytechnique Fédérale de Lausanne, Switzerland). Viral particles were produced by transient co-transfection of 293T cells with the recombinant pLVTHM lentivector constructs, the packaging vector psPAX2 and the envelope vector pMD2.G. Ep5ExTu cells were then transduced with the lentiviral particles contained in supernatants of transfected 293T cells, and stable cell lines were selected by fluorescence-activated cell sorting (FACS) on the basis of green fluorescent protein (GFP) expression. Clones of Ep5ExTu cells expressing Sh-VE-cadherin and Sh-N-cadherin were expanded and the expression of VE-cadherin and N-cadherin was monitored by immunoblot. Experiments were approved by the Sächsisches Staatsministerium für Umwelt und Landwirtschaft, Dresden, Germany (Re: 55-8811.72/69).

### Silencing VE-cadherin or N-cadherin in human breast cancer cell lines

Human SUM 149 cells were cultured in DMEM-F12 supplemented with 15% FCS. Silencing of human N-cadherin or human VE-cadherin was performed by using SMARTpools (Dharmacon, Lafayette, CO, USA). Cells (1-2 × 10^5^) were seeded in 2 ml DMEM, 15% FCS in 6-well plates 24 h before transfection. The medium was replaced by 2 ml Opti-MEM I (Invitrogen, Karlsruhe, Germany) 1 h before transfection. 100 pmol siRNA was mixed with 500 μl Lipofectamine 2000 (Invitrogen) diluted in a final volume of 1 ml Opti-MEM I and incubated for 30 min at room temperature to allow the formation of complexes. For transfection, the medium was removed and the DNA-Lipofectamine mixture was added to the cells, which were then incubated at 37°C. 1 ml DMEM, 15% FCS was added 6 h after transfection and the cells were cultivated for another 24 h before analysis.

### Generation of Sh-N-cadherin cell lines stably expressing VE-cadherin

Mouse cDNA encoding VE-cadherin (kindly provided by Prof. D. Vestweber, Münster, Germany) was cloned into the P6NST50 vector (kindly provided by Prof. D. Lindemann, Dresden, Germany). Ep5ExTu (Sh-N-cad2) cells were transduced with the VE-cadherin-encoding virus particles. Cells stably expressing VE-cadherin were then selected by FACS on the basis of their GFP expression.

### Cell proliferation

Ep5ExTu cells (10^5^) were plated and labeled with bromodeoxyuridine (BrdU) in 96-well plates in DMEM-F12 supplemented with 15% FCS. After 24 h, cell proliferation was quantified using a colorimetric immunoassay based on BrdU incorporation according to manufacturer's instructions (Roche, Mannheim, Germany). The amount of BrdU incorporated into the cells was measured by using an ELISA plate reader (Plus MS2 Reader, Titertek, Huntsville, AL, USA). For each independent experiment, six wells per condition were used.

### RNA isolation and reverse transcription-PCR analysis

Ep5ExTu cells (2 × 10^5^) were seeded in 4 ml DMEM-F12 supplemented with 15% FCS in 6 cm dishes 24 h before RNA isolation. Total RNA was isolated from cell lysates using a universal RNA Purification Kit according to the manufacturer's protocol (Roboklon, Berlin, Germany). Aliquots of 3 μg of total RNA were reverse transcribed using Superscript II (Invitrogen, Karlsruhe, Germany) and random hexameric primers (Roche, Mannheim, Germany). The sequences of the PCR primers used are shown in Additional file 1. The intensity of the PCR bands was quantified using Bio-Rad densitometer and Quantity One analysis software (Hercules, CA, USA). qRT-PCR analysis for human VE-cadherin and N-cadherin was performed following reverse transcription of total RNA using a Reverse Transcriptase Core Kit (Eurogentec, Seraing, Belgium), by real-time PCR (Mastercycler ep Realplex; Eppendorf, Hamburg, Germany) using QuantiFast SYBR Green PCR Kit (Qiagen, Valencia, CA, USA). All reactions were run in duplicates and Ct values were normalized against the GAPDH gene, using the delta-delta-Ct method. Primer sequences used were: VE-cadherin (CDH5), (forward) 5'-CGT GAG CAT CCA GGC AGT GGT AGC-3', (reverse) 5'-GAG CCG CCG CCG CAG GAA G-3'; N-cadherin (CDH2) (forward) 5'-CCA CCT TAA AAT CTG CAG GC-3', (reverse) 5'-GTG CAT GAA GGA CAG CCT CT-3'; GAPDH, (forward) 5'-CTC CTC TGA CTT CAA CAG CGA CA-3', (reverse) 5'-GAG GGT CTC TCT CTT CCT CTT GT-3'.

### Immunoblot analysis

Immunoblot analysis was performed as described previously [7,26,27]. The primary antibodies used were anti-VE-cadherin (R&D Systems, Wiesbaden, Germany), anti-N-cadherin and anti-β-actin (Sigma-Aldrich, Munich, Germany). The secondary antibodies were horseradish peroxidase-conjugated anti-rabbit immunoglobulin G (IgG) (Novus Biologicals, Littleton, CO, USA), anti-goat IgG (Jackson Immunoresearch, Soham, UK) and anti-mouse IgG (Cell Signaling Technology, Frankfurt, Germany). Band intensity was quantified using Quantity One analysis software (Bio-Rad, Hercules, CA, USA). Antibodies used for detection of human VE-cadherin, N-cadherin and E-cadherin were goat anti-VE-cadherin (Santa Cruz Biotechnology, Santa Cruz, CA, USA), mouse anti-N-cadherin (BD Biosciences, Bedford, MA, USA), mouse anti-E-cadherin (BD Biosciences, Bedford, MA, USA). Membranes were incubated with secondary antibody conjugated with IRDye Infrared Dyes (IRDYE 800CW donkey anti-goat, IRDYE 800CW donkey anti-mouse), and bands were revealed with a LI-COR scanner (LI-COR Biosciences, Lincoln, NE, USA).

### Immunofluorescence staining of cells

Cells (2 × 10^5^) were seeded on glass coverslips. After 24 h, cells were fixed as described [7] and stained with the following primary antibodies: anti-VE-cadherin (R&D Systems, Wiesbaden, Germany), anti-N-cadherin (BD Biosciences, Bedford, MA, USA), anti-E-cadherin (Sigma-Aldrich, Munich, Germany), anti-β-catenin (Cell Signaling Technology, Frankfurt, Germany) and anti-Vimentin (Sigma-Aldrich, Munich, Germany). The secondary antibodies used were goat anti-rat Alexa 594, chicken anti-rabbit Alexa 594 and rabbit anti-goat 594 (Molecular Probes, Leiden, The Netherlands). Antibodies used for detection of human VE-cadherin, N-cadherin and E-cadherin were: goat anti-VE-cadherin (Santa Cruz Biotechnology, Santa Cruz, CA, USA), mouse anti-N-cadherin (BD Biosciences, Bedford, MA, USA), mouse anti-E-cadherin (BD Biosciences, Bedford, MA, USA).

### Tumor experiments

Ep5ExTu cells were cultured in DMEM-F12 supplemented with 15% FCS. Cells were then trypsinized, rinsed twice in 5 ml PBS and resuspended in PBS at a concentration of 1 × 10^6 ^cells/ml. Tumor experiments were performed as described previously [27] with eight- to twelve-week-old female BALB/c mice (Taconic, Ejby, Denmark). All tumors were measured with calipers every two to three days and the volume of each measurement was calculated as: (width^2 ^× length)/2. Tumors were collected 10 or 15 days after injection, embedded in Tissue Tek (Sakura Finetek, Staufen, Germany) and frozen on dry ice. Animal experimentation was approved by the Landesdirektion Dresden, Germany (Re: 24-9168.11-1/2009-19).

### Immunofluorescence staining of frozen tumor sections

Eight μm frozen sections were cut and air dried. Sections were then fixed in 100% acetone for 10 min at -20°C and air dried. Rehydrated sections were stained with antibodies as described for immunofluorescence staining of cells.

### Immunohistochemistry on human tumor tissue microarray

The tissue microarray included formalin-fixed, paraffin-embedded probes of 84 invasive breast cancers. The clinicopathological features are summarized in Additional file 2. Specimens were dewaxed, and immunohistochemical staining was performed using an automated immunostainer according to the manufacturer's protocol (Benchmark; Ventana Medical Systems, Tucson, AZ, USA) as described previously [7]. The primary antibodies were anti-human VE-cadherin and anti-human N-cadherin (Polyclonal; Abcam, Cambridge, UK). The signal was amplified using the VENTANA amplification kit (Benchmark; Ventana Medical Systems, Tucson, AZ, USA) and visualized using avidin-biotin labeling and 3, 3'-diaminobenzidine. Slides were counterstained with hematoxylin. Evaluation of the staining was performed separately for nuclear, cytoplasmic and membrane-associated expression in a semi-quantitative manner. Expression was considered as positive if at least 1% of the tumor cells were stained. Chi-squared test was used for statistical evaluation of the results, and a *P *value < 0.05 was considered as statistically significant. The study was approved by the Ethics Committee (Ethikkommisssion) of the Faculty of Medicine of the University of Dresden (Re: EK59032007).

## Results

### N-cadherin and VE-cadherin are co-expressed in human breast cancer tissue

**Figure 1:**
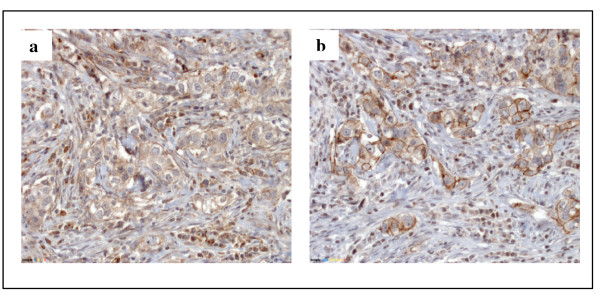
**Immunohistochemical staining of vascular endothelial (VE)-cadherin (a) and neural (N)-cadherin (b) in human breast cancer tissue**.

We recently reported that VE-cadherin is aberrantly expressed in approximately 73% of human invasive mammary carcinomas (27 out of 37 cases). Expression was observed in a subset of tumor cells, as well as in the tumor vasculature. Here, we have extended this analysis by examining a larger number (*n *= 84) of invasive breast cancer specimens in a tissue microarray. This array included carcinomas of different tumor stages, histological types and histopathological grades. The clinicopathological features are summarized in Additional file 2. VE-cadherin protein was detected by immunohistochemistry in a subset of tumor cells in 61 of the 79 (77%) invasive carcinomas examined (Table 1). Yet, we did not observe a significant correlation between VE-cadherin expression and clinicopathological factors (data not shown). 82% of the tumors expressed N-cadherin protein, and 62% of the specimens were positive for both VE- and N-cadherin. This result shows for the first time that these two cadherins can be co-expressed in breast cancer (Figure 1). However, the localization of VE- and N-cadherin in tumor cells appears to be partially different (Table 1). VE-cadherin membrane staining was observed in 31 tumors (39%) whereas N-cadherin was localized at the membrane in only 6 breast cancers (7%). Table 2 summarizes the relationship between N-cadherin expression and the clinicopathological features of the breast cancers: membrane localization of N-cadherin in tumors significantly correlated with smaller tumor size (5 out of 36 cases pT1 vs. 1 out of 47 cases pT2, *P *= 0.04); in contrast, nuclear expression of N-cadherin was observed primarily in poorly differentiated tumors.

**Table 1:** Neural (N)-cadherin and vascular endothelial (VE)-cadherin immunoreactivity in human mammary carcinoma cells.

N-cadherin expression	Positzive	Negative	Positive expression percentage
Membrane	6	78	7%

Cytoplasmic	29	55	34%

Nuclei	55	29	65%

Total expression	69	15	82%

**VE-cadherin expression**	**Positive**	**Negative**	**Positive expression percentage**

Membrane	31	48	39%

Cytoplasmic	56	23	70%

Total expression	61	18	77%

**Table 2:** Correlation between neural (N)-cadherin expression and clinicopathological factors.

	N-cadherin membrane expression		
**Parameter**	**Negative**	**Positive**	***P *value**

Tumor size (pT)			0.040
1 (Tumor size ≤ 2 cm)	31	5	
2 (Tumor size > 2 cm)	46	1	

	**N-cadherin nuclear expression**		

Parameter	Negative	Positive	*P *value

Histopathological Grading			0.006
1	7	0	
2	15	25	
3	13	22	

### N-cadherin knockdown results in reduction of VE-cadherin expression in breast cancer cells

**Figure 2:**
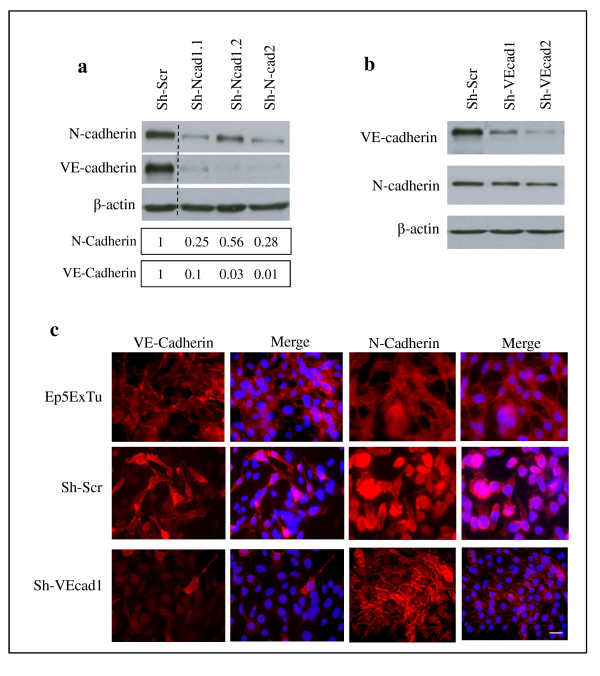
**Neural (N)-cadherin regulates vascular endothelial (VE)-cadherin expression in Ep5ExTu cells**. **(a) **N-cadherin silencing in Ep5ExTu cells transduced either with a scrambled shRNA-containing virus (Sh-Scr) or with Sh-N-cadherin virus (Sh-N-cad1.1, Sh-N-cad1.2 and Sh-N-cad2). The bars represent the relative N-cadherin or VE-cadherin protein levels in Sh-N-cadherin cell lines versus the Sh-Scr cell line, as determined by western blot analysis. **(b) **VE-cadherin protein expression was examined by western blot in Ep5ExTu cells that were transduced with the VE-cadherin shRNA (Sh-VE-cad-1 and Sh-VE-cad-2) and in the Sh-Scr cell lines. β-actin was detected as loading control. **(c) **Immunofluorescence staining for VE-cadherin or N-cadherin in Sh-VE-cad-1 and Sh-Scr cells. Ep5ExTu cells were used as a positive control for the localization of VE-cadherin and N-cadherin. Nuclear staining with DAPI is also shown. Bar, 60 μm.

To investigate the functional role of N-cadherin in breast cancer cells, N-cadherin expression was silenced by transfection of two different siRNAs into Ep5ExTu cells. Unexpectedly, downregulation of N-cadherin in Ep5ExTu cells resulted in reduced VE-cadherin expression (see Additional file 3). Next, we stably silenced N-cadherin expression by lentiviral transduction with two different short hairpin RNAs (shRNAs), Sh-Ncad1 and Sh-Ncad2. We selected three independent cell clones (Sh-Ncad1.1, Sh-Ncad1.2 and Sh-Ncad2) that displayed reduced N-cadherin expression in comparison to the control cell line that had been transduced with a scrambled shRNA (Sh-Scr) (Figure 2a). In Sh-Ncad1.1 and Sh-Ncad2 cells, N-cadherin protein levels were reduced by approximately 75% as compared to the Sh-Scr cell line, whereas in Sh-Ncad1.2 cells, N-cadherin protein expression was reduced by only 45%. Consistent with the results obtained from the transient transfection with siRNA (see Additional file 3), immunoblot analysis (Figure 2a) and immunofluorescence staining (see Additional file 3) of the cells revealed also a clear reduction in VE-cadherin expression in the N-cadherin knockdown cell lines (Sh-Ncad1.1, Sh-Ncad1.2 and Sh-Ncad2). This result suggests that N-cadherin is required for sustaining the expression of VE-cadherin in Ep5ExTu cells.

In contrast, VE-cadherin silencing by lentiviral transduction of Ep5ExTu cells with shRNA specific for VE-cadherin affected the protein level of N-cadherin only slightly because N-cadherin levels were similar to the Sh-VE-cadherin cell lines (Sh-VEcad1 and Sh-VEcad2) and the control Sh-Scr cell line (Figure 2b). However, as described for endothelial cells [28,29], VE-cadherin expression affected N-cadherin localization (Figure 2c and [7]). In the Sh-Scr cell line, VE-cadherin was expressed heterogeneously and presented at the cell surface similar to untransfected Ep5ExTu cells, whereas N-cadherin staining was uniformly distributed over the cell and localized at cell-cell contacts in only a few cells (Figure 2c). In cells silenced for VE-cadherin (Sh-VEcad1), N-cadherin was enriched at cell-cell contacts. This shows that silencing of VE-cadherin leads to a redistribution of N-cadherin to cell-cell contacts, without influencing its expression levels significantly.

We also investigated the expression of VE-cadherin and N-cadherin in the human breast carcinoma cell line, SUM 149. By western blot analysis, immunofluorescence staining and qRT-PCR, SUM 149 cells expressed VE-cadherin and N-cadherin, in addition to E-cadherin (see Additional File 4). Knockdown of N-cadherin resulted in reduction of VE-cadherin mRNA levels but not vice versa. Thus, regulation of VE-cadherin by N-cadherin is observed in both mouse and human breast cancer cells.

### Suppression of N-cadherin in mesenchymal Ep5ExTu cells induces MET and re-expression of E-cadherin

**Figure 3:**
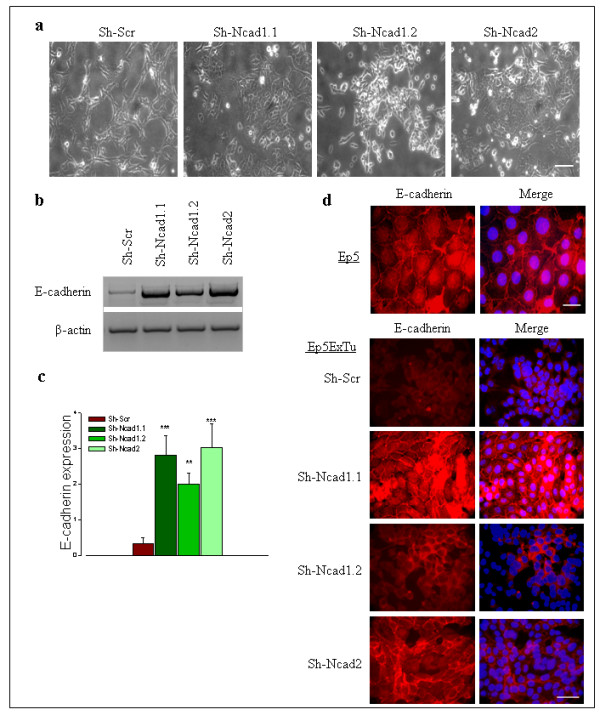
**Neural (N)-cadherin silencing in Ep5ExTu cells induced epithelial (E)-cadherin expression and influenced its localization in the cells in a dose-dependent manner**. **(a) **Phase contrast microscopy of control cell lines (Sh-Scr) and Sh-N-cadherin cell lines (Sh-N-cad1.1, Sh-N-cad1.2 and Sh-N-cad2). Bar, 100 μm. **(b) **E-cadherin mRNA levels in Ep5ExTu clones. **(c) **The graph represents the quantification of the relative amount of E-cadherin mRNA in three independent experiments. β-actin was used as loading control. The mean ± SD is indicated. (*n *= 3) (**, *P *< 0.0012 and ***, *P *< 0.0009). **(d) **Immunolocalization of E-cadherin in Ep5 (Bar, 30 μm) and Ep5ExTu (Bar, 60 μm) clones by immunofluorescence microscopy. Nuclear staining with DAPI is also shown.

Ep5ExTu cells exhibit fibroblastoid characteristics, such as elongated shape and relatively weak cell-cell contacts as compared to epithelial cells [7,25,26]. We have previously observed that E-cadherin is expressed in epithelial Ep5 cells, but not in fibroblastoid Ep5ExTu cells, in agreement with the observation that E-cadherin is downregulated during EMT [7]. The two Ep5ExTu cell lines in which N-cadherin expression was efficiently silenced by shRNA (Sh-Ncad1.1 and Sh-Ncad2), displayed a cobblestone-like appearance characteristic for epithelial cells, with individual cells abutting each other (Figure 3a). Consistent with the idea that these cells have undergone MET, the epithelial phenotype of these Sh-N-cadherin cell lines was associated with re-expression of E-cadherin (Figure 3b and 3c). Thus, E-cadherin can be re-induced in Ep5ExTu cells by blocking N-cadherin expression. Interestingly, E-cadherin expression was stronger in the Sh-Ncad1.1 and Sh-Ncad2 cell lines than in the Sh-Ncad1.2 cell line (Figure 3b and 3c), in which N-cadherin expression is only moderately suppressed. Similar to Ep5 cells, E-cadherin staining was observed at sites of cell-cell contacts in both Sh-Ncad1.1 and Sh-Ncad2 cell lines (Figure 3d). These results suggest that the re-induction of E-cadherin in Ep5ExTu cells and its localization at cell-cell contacts correlates with the extent of N-cadherin reduction: the lower the N-cadherin levels are, the more pronounced is the epithelial phenotype. Silencing VE-cadherin in Ep5ExTu cells, in contrast, led to only a partial gain of the epithelial morphology and was not associated with re-induction of E-cadherin (see Additional file 5). This shows that N-cadherin has a more pronounced influence on the maintenance of the mesenchymal tumor cell phenotype than VE-cadherin.

### N-cadherin silencing influences β-catenin localization and the expression of EMT regulator genes in Ep5ExTu cells

**Figure 4:**
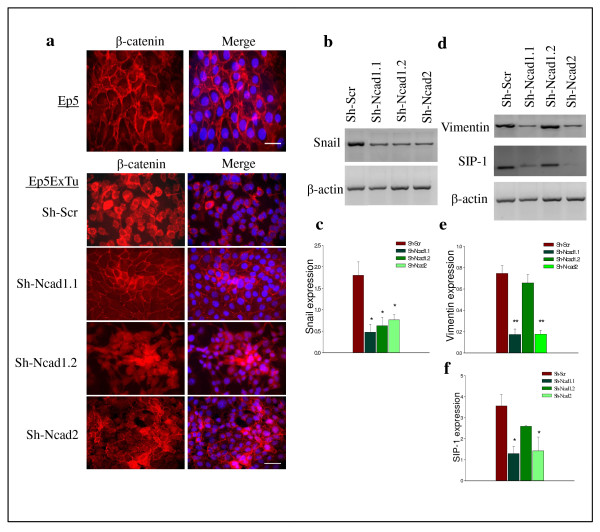
**Significant suppression of neural (N)-cadherin in Ep5ExTu cells influences β-catenin localization and the level of epithelial-mesenchymal transition (EMT) regulator genes in Ep5ExTu cells**. **(a) **Immunolocalization of β-catenin in the Sh-Scr cell line and Sh-N-cadherin cell lines (Sh-N-cad1.1, Sh-N-cad1.2 and Sh-N-cad2). Ep5 cells were used as a positive control for the localization of β-catenin in epithelial cell lines that have not undergone EMT. Bar, 30 μm. **(b) **and **(c) **mRNA level of Snail, vimentin and Smad interacting protein 1 (SIP1) in Ep5ExTu clones. **(d)**, **(e) **and **(f) **the bars indicate the relative expression levels of snail, vimentin and SIP1 mRNA in three independent experiments. β-actin was used as loading control. The mean ± SD is (*, *P *< 0.0491 and **, *P *< 0.0014) (*n *= 3).

β-catenin accumulation in the nuclei of carcinoma cells and transcriptional activation of its target genes are known to take place during malignant transformation, thereby stimulating tumor progression [2,30-33]. E-cadherin can sequester β-catenin in epithelial cells, thus lowering the amount of cytoplasmic and nuclear β-catenin, decreasing its transcriptional activity and inhibiting its tumorigenic potential [34]. To investigate whether the localization of β-catenin in Ep5ExTu breast cancer cells is altered by N-cadherin silencing, we performed immunofluorescence staining. In (epithelial) Ep5 cells, β-catenin was localized at the plasma membrane (Figure 4a) where also E-cadherin is expressed (Figure 3d). Likewise, β-catenin was localized at the plasma membrane along with E-cadherin in the two Sh-N-cadherin cell lines that had greatly reduced N-cadherin (Sh-Ncad1.1 and Sh-Ncad2) (Figure 3d, Figure 4a). In contrast, clone Sh-Ncad1.2, which had moderately reduced N-cadherin, and clone Sh-VE-cadherin showed no significant differences in β-catenin localization compared to the Sh-Scr control cell line (Figure 4a and Additional file 6), and we observed localization of β-catenin to cell-cell contacts in only a few cells. Together, these results show that efficient silencing of N-cadherin in mesenchymal murine mammary carcinoma cells leads to the re-expression of E-cadherin and the co-localization of this receptor with β-catenin at the plasma membrane.

Nuclear localization of β-catenin and the expression of vimentin are involved in epithelial-to-mesenchymal transition and correlate with enhanced invasive and migratory properties of cells [24,35]. Because β-catenin can stimulate vimentin expression [36], we tested whether the recruitment of β-catenin to the cell membrane of the N-cadherin knockdown cell lines is correlated with changes in the expression of EMT-related genes. The expression of the transcription factor Snail was reduced in all Sh-N-cadherin cell lines as compared to the control cell line (Figure 4b and 4d), whereas the expression of Smad interacting protein-1 (SIP1) and vimentin, which are involved in EMT [37], was downregulated only in Sh-Ncad1.1 and Sh-Ncad2 cell lines (Figure 4c-f and Additional file 6). Whether or not vimentin and SIP1 expression is regulated by N-cadherin remains to be determined.

### Efficient silencing of N-cadherin in Ep5ExTu cells inhibits tumor growth *in vivo *without affecting cell proliferation *in vitro*

**Figure 5:**
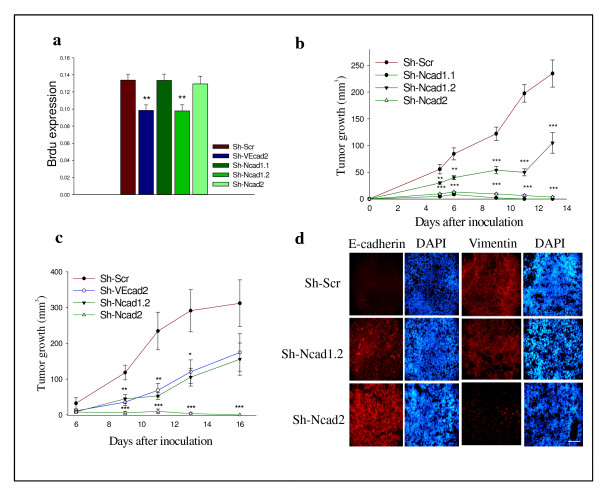
**The efficient suppression of neural (N)-cadherin in Ep5ExTu cells inhibits tumor growth *in vivo *without affecting cell proliferation *in vitro***. **(a) **Twenty-four hours after seeding equal numbers of cells, cell proliferation was quantified by measuring bromodeoxyuridine (BrdU) incorporation. The mean ± SD is (**, *P *< 0.0010) (*n *= 12). **(b) **Sh-N-cadherin cell lines (Sh-N-cad1.1, Sh-N-cad1.2 and Sh-N-cad2) or Sh-Scr were injected subcutaneously into BALB/c mice. (**, *P *< 0.0075 and ***, *P *< 0.0001) (*n *= 10). **(c) **Sh-N-cadherin cell lines (Sh-N-cad1.2 and Sh-N-cad2), Sh-VE-cadherin cell line (Sh-VE-cad-2) or Sh-Scr cell line injected into BALB/c mice. The mean ± SD is indicated (*, *P *< 0.334; **, *P *< 0.0083; ***, *P *< 0.0001) (*n *= 8). **(d) **Immunofluorescence staining for epithelial (E)-cadherin and vimentin on tumor sections. Nuclear staining with DAPI is also shown. Bar, 100 μm.

We have previously shown that silencing VE-cadherin leads to reduced Ep5ExTu cell proliferation and tumor growth [7]. To determine if N-cadherin downregulation has a similar effect, we evaluated the proliferation of Sh-N-cadherin cell lines *in vitro *by BrdU staining. Sh-VEcad2 and Sh-Ncad1.2 cells displayed lower proliferation rates than the control cell line, whereas the proliferation rate of Sh-Ncad1.1 and Sh-Ncad2 cells was not reduced (Figure 5a). To study the effect of N-cadherin on tumor growth, each of the three Sh-N-cadherin cell lines and the control cell line (Sh-Scr) were injected subcutaneously into BALB/c mice. All Sh-N-cadherin cell lines grew significantly slower than the control cell line (Figure 5b). Notably, the Sh-Ncad1.1 and Sh-Ncad2 cell lines, which showed the strongest reduction of N-cadherin expression, hardly grew *in vivo*. The Sh-Ncad1.2 cell line showed an intermediate growth rate, similar to the Sh-VE-cadherin cell line (Figure 5c). These data show that a strong (approximately 75%) reduction of N-cadherin expression in Ep5ExTu cells is necessary to inhibit tumor growth efficiently. To perform a histological analysis of tumor tissue, we isolated tumors at day 10 post injection. Downregulation of N-cadherin and VE-cadherin expression in the Sh-Ncad1.2 and Sh-Ncad2 tumors was confirmed by immunofluorescence staining (see Additional file 7). E-cadherin staining was only visible in the Sh-N-cadherin tumors (Figure 5d), and was more intense in Sh-Ncad2 than in Sh-Ncad1.2 tumors. Because vimentin expression has been implicated in carcinogenesis [38,39], we also examined the expression of this intermediate filament in the different experimental tumor groups. Vimentin expression was strongly reduced only in the Sh-Ncad2 cell line (Figure 5d). Taken together, our results suggest that both re-induction of E-cadherin expression and downregulation of vimentin expression contribute to tumor growth inhibition resulting from N-cadherin silencing in Ep5ExTu cells.

### Forced expression of VE-cadherin in N-cadherin deficient tumor cells does not enhance cell proliferation *in vitro *or tumor growth

**Figure 6:**
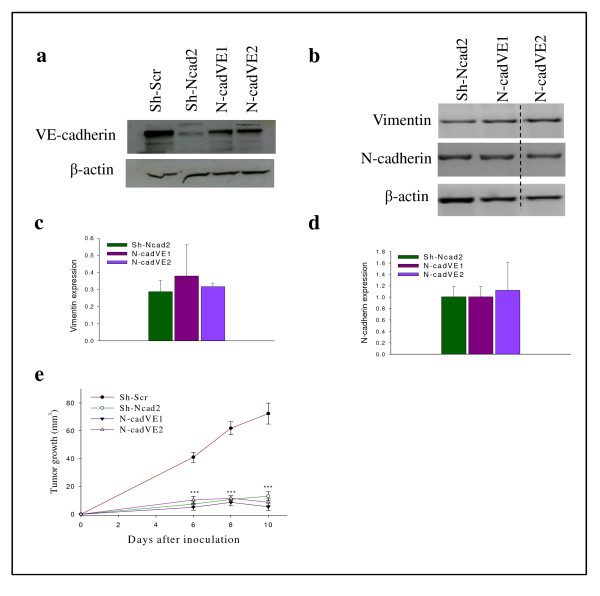
**Forced expression of vascular endothelial (VE)-cadherin in Sh-N-cad18-4 cell line does not accelerate tumor growth *in vivo***. **(a) **Western blot analysis for VE-cadherin in Sh-N-cadherin (Sh-N-cad2), Sh-N-cad2-expressing VE-cadherin cell lines (N-cadVE-1 and N-cadVE-2), and Sh-Scr. **(b) **Vimentin and neural (N)-cadherin mRNA expression levels in Ep5ExTu clones. Diagrams in **(c) **and **(d) **depict the quantification of the relative amount of vimentin and N-cadherin mRNA as determined in three independent experiments. β-actin was used as loading control. **(e) **Sh-N-cadherin (Sh-N-cad2), Sh-N-cad2-expressing VE-cadherin cell lines (N-cadVE-1 and N-cadVE-2) or Sh-Scr cell lines were injected into BALB/c mice. The mean ± SD is given (***, *P *< 0.0001) (*n *= 12).

Because N-cadherin silencing in Ep5ExTu cells also led to a reduction of VE-cadherin expression, and VE-cadherin silencing inhibited tumor growth, we wanted to determine whether the loss of VE-cadherin contributes to the tumor growth inhibition resulting from silencing N-cadherin. To address this question, we determined whether the forced expression of VE-cadherin in Sh-Ncad2 cells can affect tumor growth of N-cadherin-deficient cancer cells. A lentiviral expression vector encoding VE-cadherin was therefore introduced into Sh-Ncad2 cells. Two independent clones (N-cadVE1 and N-cadVE2) showed VE-cadherin expression levels similar to that of the Sh-Scr cell line (Figure 6a). The forced expression of VE-cadherin had no influence on cell morphology (see Additional file 8), and the expression of N-cadherin and vimentin in N-cadVE1 and N-cadVE2 cells remained unchanged (Figure 6b). These results suggest that expression of VE-cadherin in the absence of N-cadherin cannot induce Sh-Ncad2 cells to undergo EMT.

Next, we explored whether forced expression of VE-cadherin can influence cell proliferation. No significant difference in cell proliferation was observed between the VE-cadherin-expressing cell lines (N-cadVE1 and N-cadVE2) and control cell line (Sh-Ncad2) (see Additional file 8). To evaluate whether forced VE-cadherin expression influences the growth of Sh-N-cad2 tumors, we inoculated cells from the VE-cadherin-expressing lines (N-cadVE1 and N-cadVE2), Sh-N-cad2 or control Sh-Scr cell lines into wild-type BALB/c mice and monitored tumor growth. The VE-cadherin-expressing cell lines (N-cadVE1 and N-cadVE2) and Sh-Ncad2 displayed comparable growth rates and scarcely grew *in vivo *(Figure 6c). These results show that forced VE-cadherin expression cannot accelerate tumor growth in the absence of N-cadherin expression, indicating that the concomitant loss of VE-cadherin expression does not contribute to the reduction of tumor growth resulting from N-cadherin silencing.

### Forced VE-cadherin expression in Ep5ExTu cells does not affect E-cadherin localization

**Figure 7:**
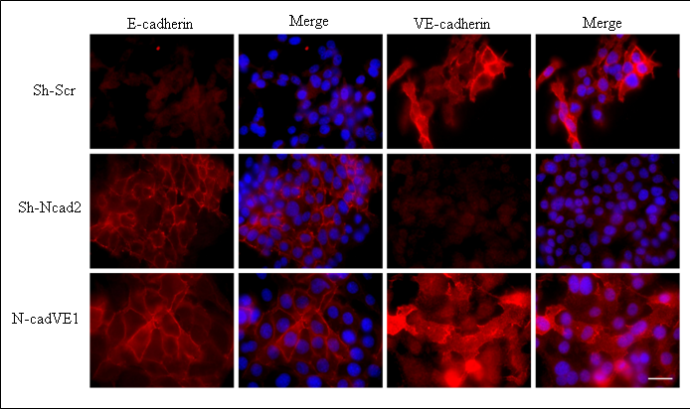
**Vascular endothelial (VE)-cadherin expression in Sh-N-cad2 has no influence on epithelial (E)-cadherin localization**. Immunofluorescence staining for E-cadherin and VE-cadherin in the Sh-N-cadherin cell line (Sh-N-cad2) and in Sh-N-cad2 cell lines expressing VE-cadherin (N-cadVE-1). Ep5ExTu cells were used as positive control for VE-cadherin and neural (N)-cadherin localization. Nuclear staining with DAPI is also shown. Bar, 30 μm.

Since the localization of VE-cadherin within adherens junctions is required for its function [5], we examined the possibility that the inappropriate localization of VE-cadherin is responsible for its inability to restore tumor growth of N-cadherin-deficient cells. By immunofluorescence staining, VE-cadherin expression was observed at the cell borders in N-cadVE1 cells, similar to control Sh-Scr cells (Figure 7). E-cadherin was also localized at cell-cell junctions as in the Sh-Ncad2 control cell line (Figure 7). This shows that VE-cadherin cannot displace E-cadherin from the cancer cell surface.

## Discussion

Breast cancer is one the leading causes of death due to cancer worldwide. Although the genetic defects underlying breast carcinogenesis have been extensively studied, important signaling pathways involved in the progression of this specific tumor type are still poorly characterized. The loss of E-cadherin and concomitant gain of N-cadherin expression is known to promote EMT and carcinoma progression. Our previous observation that endothelial cell-selective VE-cadherin is expressed aberrantly in breast cancer cells and promotes their proliferation both *in vitro *and *in vivo *[7] led us to analyze the specific roles of these cadherins as well as their interplay in experimental breast cancer in more detail. Here, we show that N-cadherin silencing in murine breast cancer cells suppresses tumor growth by upregulating E-cadherin, repressing EMT regulators, and reversing the invasive mesenchymal phenotype to epithelial phenotype. Although both N-cadherin and VE-cadherin promote tumor growth, their influence on E-cadherin expression in mesenchymal tumor cells is divergent: whereas N-cadherin is capable of repressing E-cadherin expression in Ep5ExTu cells, VE-cadherin has no effect on its expression levels [7]. Moreover, N-cadherin is required for maintaining VE-cadherin expression, but not vice versa. The regulation of VE-cadherin expression by N-cadherin is a novel mechanism of tumor progression in breast cancer and shows that N-cadherin both inhibits the expression of E-cadherin and stimulates the expression of VE-cadherin.

The downregulation of VE-cadherin in the N-cadherin-deficient Ep5ExTu cells shows that N-cadherin is required (although not necessarily sufficient) for VE-cadherin expression in aggressive carcinoma cells. Regulation of VE-cadherin by N-cadherin was already described before in (nonmalignant) human umbilical vein endothelial cells (HUVEC) [40], however, evidence for direct regulation of VE-cadherin by N-cadherin is lacking, and the precise mechanisms involved in this regulation remain to be determined. The ability of N-cadherin to regulate VE-cadherin was nonreciprocal because VE-cadherin silencing had no effect on N-cadherin expression. However, as described also for other cell types [28,29], VE-cadherin expression in Ep5ExTu cells affected the localization of N-cadherin protein. In control Ep5ExTu cells, which express both cadherins, N-cadherin displayed a nonjunctional distribution whereas in Sh-VE-cadherin knockdown cell lines, N-cadherin was enriched at cell-cell junctions. Whether VE-cadherin expression can influence signaling pathways regulated by N-cadherin as a consequence of excluding it from cell contacts remains to be determined.

There is growing evidence indicating that EMT is a reversible process in cancer cells. Recently, it was hypothesized that tumor cells in metastatic sites can undergo re-differentiation and undergo MET [1,3,6]. This transition could allow metastatic cells to adapt to a new microenvironment. Re-expression of E-cadherin is a critical component of the MET [41,42]. However, little is known about the exact mechanism and biological or clinical significance of MET in cancer. Islam *et al. *reported that blocking N-cadherin expression upregulates E-cadherin expression in squamous epithelial cells [10]. Interestingly, we observed that N-cadherin silencing promoted multiple aspects of MET in Ep5ExTu cells in a concentration-dependent manner, including morphological changes, increased levels of E-cadherin and decreased levels of mesenchymal markers. In contrast, VE-cadherin silencing led only to a weaker induction of epithelial markers and had no effect on E-cadherin expression, indicating that MET is activated more efficiently by N-cadherin silencing than by VE-cadherin silencing. This difference might be explained by the difference in β-catenin localization in Sh-N-cadherin and Sh-VE-cadherin cell lines. Interestingly, in Sh-N-cadherin cell lines (Sh-Ncad1.1 and Sh-Ncad2) that displayed the most efficient N-cadherin downregulation, β-catenin was localized at the cell membrane like in the epithelial Ep5 cell line. As reported by other groups, alteration of β-catenin localization alone can be sufficient for the suppression of an invasive phenotype [24,43].

The intermediate filament vimentin is an important marker of EMT and its expression is related to the adhesion and migration properties of tumor cells [44]. A previous study using human breast cancer cells showed that accumulation of cytoplasmic or nuclear β-catenin and vimentin expression coincide [36]. Additionally, β-catenin can directly transactivate vimentin expression through its binding to the T cell factor (TCF)/lymphoid enhancer factor (LEF) 1 transcription factor family. Vimentin expression was consistently downregulated in mammary carcinoma cell lines in which β-catenin was localized at the plasma membrane (Sh-Ncad1.1 and Sh-Ncad2), but vimentin levels remained unchanged in lines that that showed cytoplasmic and/or nuclear distribution of β-catenin (Sh-Ncad1.2 and Sh-VEcad1).

Several transcriptional regulators are known to repress E-cadherin expression and thereby induce EMT. Among these, we analyzed the expression level of Snail and SIP1, which emerged as key factors regulating E-cadherin expression [45]. Whereas the level of Snail was downregulated in all Sh-N-cadherin cell lines, the level of SIP1 was decreased only in the Sh-Ncad1.1 and Sh-Ncad2 cell lines, which expressed higher E-cadherin levels. This result therefore suggests that the re-expression of E-cadherin is stimulated more efficiently if the expression of both transcriptional repressors of E-cadherin is decreased.

Deregulation of E-cadherin in breast cancer correlates with higher tumor grade and metastatic tumor cell behavior [46,47]. Also in other cell types and in animal models, E-cadherin has been shown to act as a suppressor of tumor growth and invasion [48]. In our study, suppressing N-cadherin significantly reduced Ep5ExTu tumor growth. Remarkably, Sh-Ncad1.1 and Sh-Ncad2 cell lines hardly grew *in vivo*, and mice injected with either cell line were often tumor-free 14 days after inoculation. Histological analysis of tumor sections isolated at day 10 post injection confirmed the re-expression of E-cadherin and downregulation of vimentin in Sh-Ncad2 tumors *in vivo*. Since N-cadherin silencing did not change the proliferation rate of Sh-Ncad1.1 and Sh-Ncad2 *in vitro*, it is likely that the phenotypic reversion of these cell lines, along with E-cadherin expression and associated β-catenin, leads to the inhibition of tumor growth *in vivo*. In contrast, moderate suppression of N-cadherin in Sh-Ncad1.2, which greatly inhibits VE-cadherin expression, resulted in a growth rate similar to the Sh-VEcad2 cell line. This growth inhibition correlates well with the decrease in cell proliferation observed for both cell lines *in vitro*. It is therefore possible that the growth inhibition of the Sh-Ncad1.2 cell line is caused primarily by the strong VE-cadherin suppression.

Does the introduction of VE-cadherin in N-cadherin (and consequently VE-cadherin) deficient cells restore tumor growth? The forced expression of VE-cadherin in the Sh-Ncad2 cells (that had undergone MET) did not change their epithelial phenotype, as indicated by unchanged E-cadherin and vimentin expression levels. Additionally, junctional localization of E-cadherin was preserved in VE-cadherin re-expressing cell lines. In line with the unaltered epithelial phenotype, forced expression of VE-cadherin failed to evoke a significant difference in tumor growth; the VE-cadherin-expressing and control cells had similar growth rates *in vivo*. These results suggest that VE-cadherin expression, at least in the presence of E-cadherin, is not sufficient to promote tumor progression.

## Conclusions

Our study shows for the first time that N-cadherin and VE-cadherin are co-expressed in human breast cancer. N-cadherin controls the expression of VE-cadherin in aggressive mouse breast cancer cells and has an important role in maintaining the mesenchymal phenotype and promoting tumor progression. VE-cadherin, on the other hand, regulates the subcellular localization of N-cadherin by displacing it from the cell surface. Our study supports the hypothesis that the interplay between cadherins in breast cancer progression is not limited to the classical 'cadherin switch', which involves the loss of E-cadherin expression or function and the gain of N-cadherin, but comprises also an intricate interdependence of N-cadherin and VE-cadherin.

## Abbreviations

BrdU: bromodeoxyuridine; DMEM: Dulbecco's modified Eagle's medium; E-cadherin: epithelial cadherin; EMT: epithelial-mesenchymal transition; FACS: fluorescence-activated cell sorting; FCS: fetal calf serum; GFP: green fluorescent protein; HUVEC: human umbilical vein endothelial cells; IgG: immunoglobulin G; MET: mesenchymal-epithelial transition; N-cadherin: neural cadherin; PBS: phosphate-buffered saline; PCR: polymerase chain reaction; shRNA: small hairpin ribonucleic acid; siRNA: small interfering RNA; SIP1: Smad interacting protein 1; TGF-β: transforming growth factor-beta; VE-cadherin: vascular endothelial cadherin.

## Competing interests

The authors declare that they have no competing interests.

## Authors' contributions

MR participated in the design of the project, performed the majority of *in vitro *experiments, analyzed the experimental tumors, and drafted the manuscript. KF helped to obtain human breast tumors and conducted tumor histology analysis. BW performed tumor experiments. AKu and AKe helped to perform *in vitro *experiments and analyses of experimental tumors. ML and GBa participated in the design of the project. HS helped to analyze expression of VE-cadherin in human cells. GB conceived the study, participated in its design and coordination and helped to draft the manuscript. All authors read and approved the final manuscript.

## Supplementary Material

Additional file 1 **List of PCR primers**. Click here for file

Additional file 2 **Histological types and histopathological grades of invasive human breast carcinomas**. Click here for file

Additional file 3 **Expression of VE-cadherin and neural (N)-cadherin in N-cadherin-silenced tumor cells**. Click here for file

Additional file 4 **Expression of classical cadherins in the human breast cancer cell line SUM 149**. Click here for file

Additional file 5 **Morphology and epithelial (E)-cadherin immunofluorescence staining of Sh-VE-cadherin and control cell lines**. Click here for file

Additional file 6 **Immunofluorescence staining of β-catenin in Sh-VE-cadherin and Sh-Ncad2 cell lines**. Click here for file

Additional file 7 **Immunofluorescence staining of neural (N)-cadherin and vascular endothelial (VE)-cadherin on tumor sections**. Click here for file

Additional file 8 **Morphology and proliferation analysis of vascular endothelial (VE)-cadherin-overexpressing Sh-Ncad2 cells**. Click here for file
