# Supplementary material for: Interplay between neural-cadherin and vascular endothelial-cadherin in breast cancer progression
Source: Breast Cancer Res. 2012 Dec 6;14(6):R154. doi: 10.1186/bcr3367 (PMC4053141; doi:10.1186/bcr3367)
Supplement: Additional file 1 — List of PCR primers. [file bcr3367-S1.PDF]

Nr	Primer	DNA-sequence(5'→3')	Temp.
1	VE-cadherin-m-forw<br>VE-cadherin-m-Rev	TTTGCCCAGCCCTACGAACCT<br>ACCGCCGTCATTGTCTGCCTC	57°C
2	N-cadherin-m-forw<br>N-cadherin-m-Rev	CGCCATCATCGCTATCCTTCTGTG<br>AGCCGCTGCCCTCGTAGTCAAA	57°C
3	E-cadherin-m-forw<br>E-cadherin-m-Rev	GAGCCTGAGTCCTGCAGTCC<br>GTATTGCTGCTTGGCCTCA	53°C
4	Snail-m-forw<br>Snail-m-Rev	GGAAGCCCAACTATAGCGAGC<br>CAGTTGAAGATCTTCCGCGAC	57°C
5	Vimentin-m-forw<br>Vimentin-m-Rev	GAAGGAAGAGATGGCTCGTC<br>CTGCACTGTTGCACCAAGTG	57°C
6	SIP-1-m-forw<br>SIP-1-m-Rev	AGGCATATGGTGACGCACAA<br>CTTGAACTTGCGGTTACCTGC	57°C

Additional file 1. List of PCR primers
