# Supplementary material for: Interplay between neural-cadherin and vascular endothelial-cadherin in breast cancer progression
Source: Breast Cancer Res. 2012 Dec 6;14(6):R154. doi: 10.1186/bcr3367 (PMC4053141; doi:10.1186/bcr3367)
Supplement: Additional file 2 — Histological types and histopathological grades of invasive human breast carcinomas. [file bcr3367-S2.PDF]

Feature	number
pT-stage	
pT1	37
pT2	35
pT3	4
pT4	8
pN-stage	
pN0	40
pN1-3	27
pNx	16
Histopathological grade (according to Elston & Ellis)	
G1	7
G2	42
G3	35
Estrogen receptor stage	
negative	18
postive	66
Progesterone receptor stage	
negative	33
positive	51
Her-2/neu stage	
negative	72
positive	9
unknown	3
Histological type	
invasive ductal	61
invasive lobular	13
other	10

Additional file 2. Histological types and histopathological grades of invasive human breast carcinomas
