# Supplementary material for: Interplay between neural-cadherin and vascular endothelial-cadherin in breast cancer progression
Source: Breast Cancer Res. 2012 Dec 6;14(6):R154. doi: 10.1186/bcr3367 (PMC4053141; doi:10.1186/bcr3367)
Supplement: Additional file 3 — Expression of VE-cadherin and neural (N)-cadherin in N-cadherin-silenced tumor cells. [file bcr3367-S3.PDF]

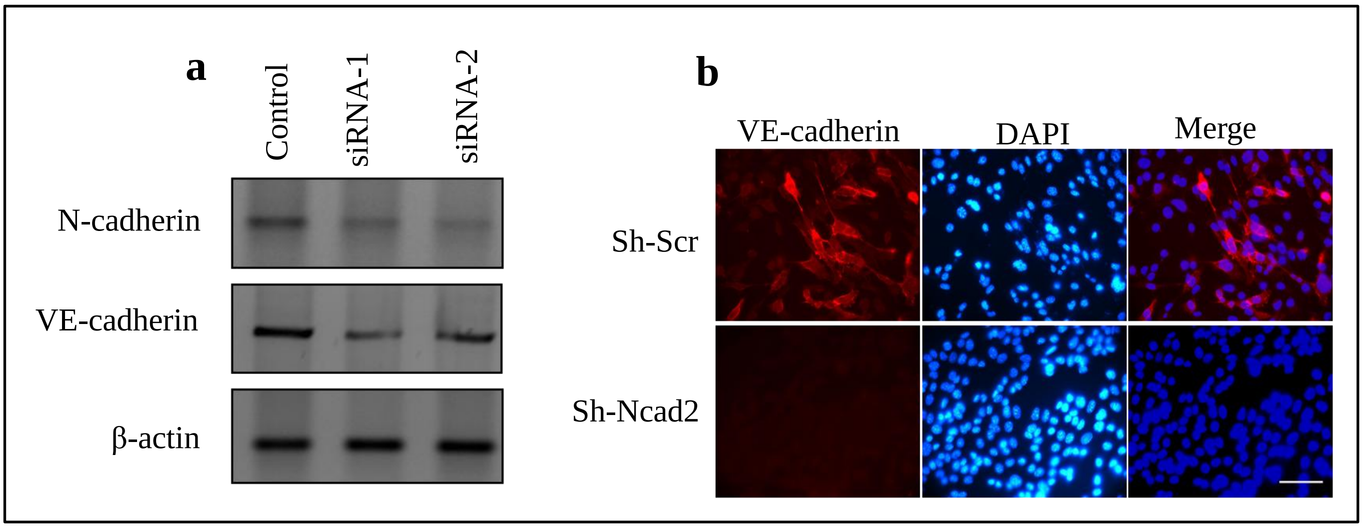

Additional file 3: (a) VE-cadherin and N-cadherin mRNA expression levels two days after transfection either with Lipofectamine 2000 only (control) or with one of two independent N-cadherin siRNA (siRNA-1 and siRNA-2). (b) Immunolocalization of VE-cadherin was determined in Scramble shRNA cell line (Sh-Scr) and N-cadherin shRNA cell line (Sh-Ncad2) by immunofluorescence staining. *Bar,* 60 µm. Nuclear staining with DAPI is depicted as well.
