# Supplementary material for: Interplay between neural-cadherin and vascular endothelial-cadherin in breast cancer progression
Source: Breast Cancer Res. 2012 Dec 6;14(6):R154. doi: 10.1186/bcr3367 (PMC4053141; doi:10.1186/bcr3367)
Supplement: Additional file 4 — Expression of classical cadherins in the human breast cancer cell line SUM 149. [file bcr3367-S4.PDF]

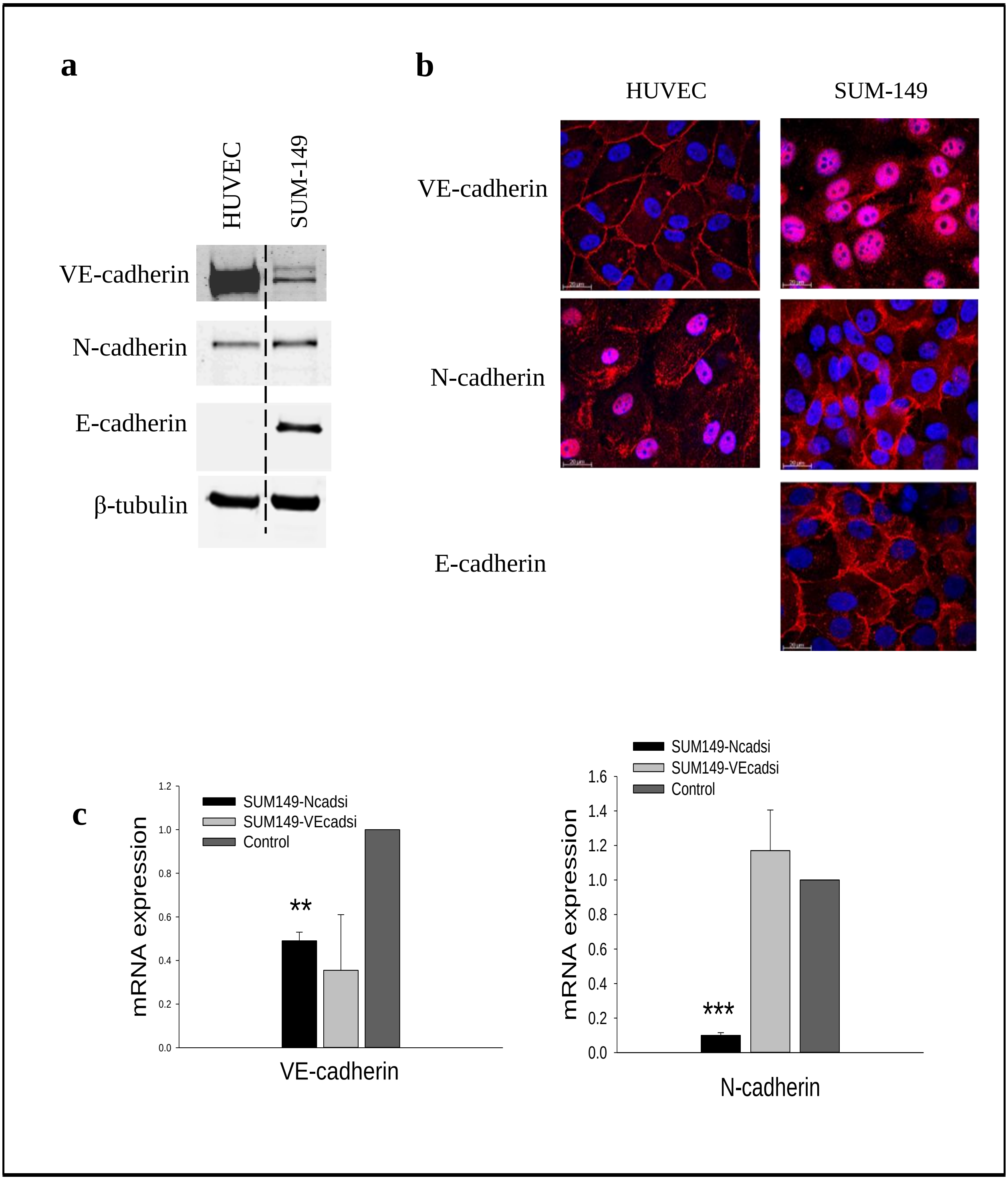

Additional File 4: Expression of classical cadherins in the human breast cancer cell line SUM 149. Western blot (a) and immunofluorescence (b) detection of VE-cadherin and N-cadherin in human breast cancer cells; human umbilical vein endothelial cells were used as a positive control for VE-cadherin. (c) As detected by qRT-PCR, knock-down of N-cadherin results in reduced VE-cadherin mRNA levels (left panel), but not vice versa. See Materials and Methods for experimental details.
