# Supplementary material for: Interplay between neural-cadherin and vascular endothelial-cadherin in breast cancer progression
Source: Breast Cancer Res. 2012 Dec 6;14(6):R154. doi: 10.1186/bcr3367 (PMC4053141; doi:10.1186/bcr3367)
Supplement: Additional file 5 — Morphology and epithelial (E)-cadherin immunofluorescence staining of Sh-VE-cadherin and control cell lines. [file bcr3367-S5.PDF]

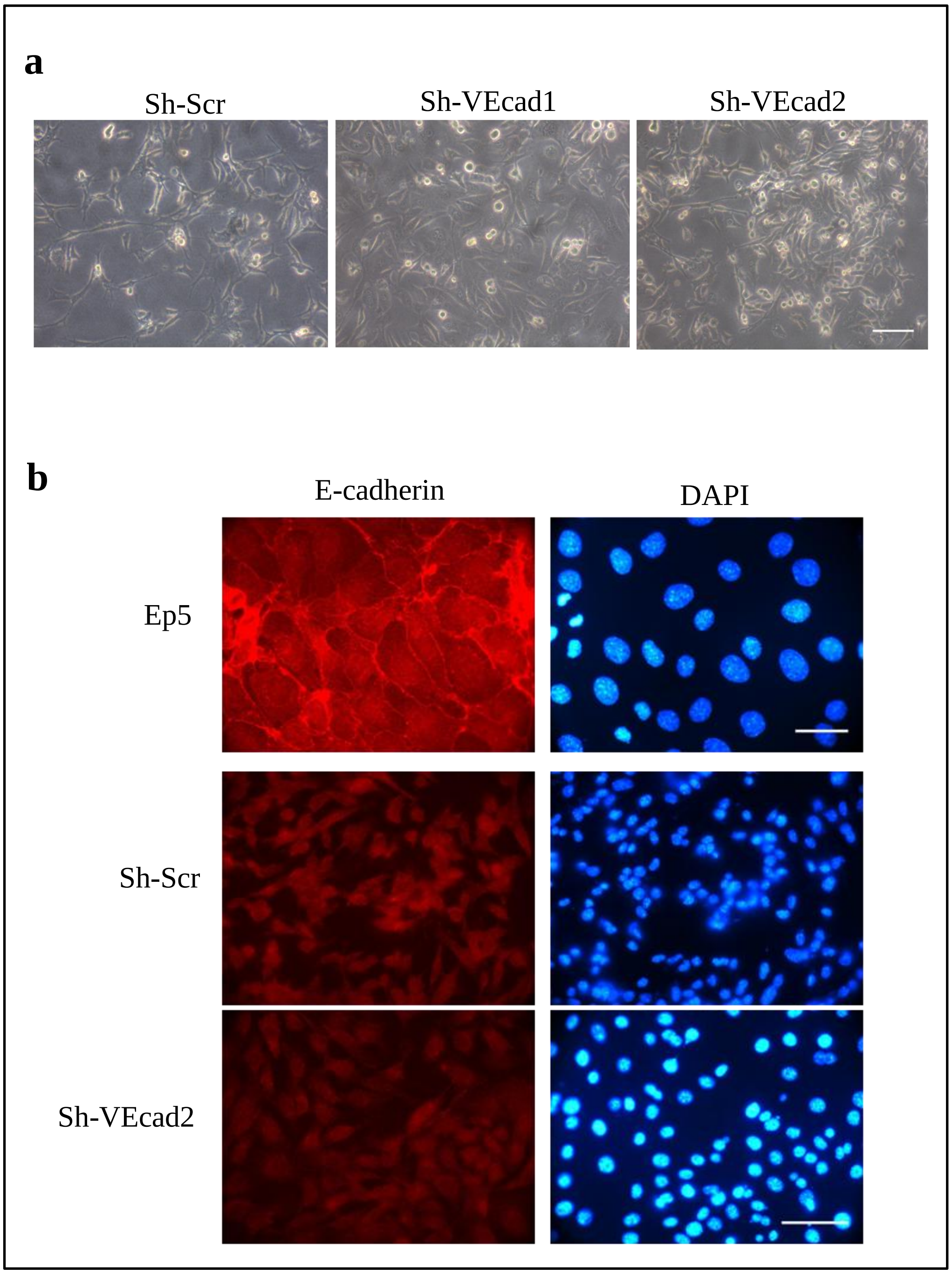

Additional file 5: (a) Phase contrast microscopy of control cell lines (Sh-Scr) and Sh-VE-cadherin cell lines (Sh-VEcad1 and Sh-VEcad2) *Bar,* 100 µm. (b) Immunofluorescence staining of E-cadherin by immunofluorescence microscopy *Bar,* 60 µm. Ep5 cells were used as a positive control for E-cadherin. *Bar,* 30 µm.
