# Supplementary material for: Interplay between neural-cadherin and vascular endothelial-cadherin in breast cancer progression
Source: Breast Cancer Res. 2012 Dec 6;14(6):R154. doi: 10.1186/bcr3367 (PMC4053141; doi:10.1186/bcr3367)
Supplement: Additional file 6 — Immunofluorescence staining of β-catenin in Sh-VE-cadherin and Sh-Ncad2 cell lines. [file bcr3367-S6.PDF]

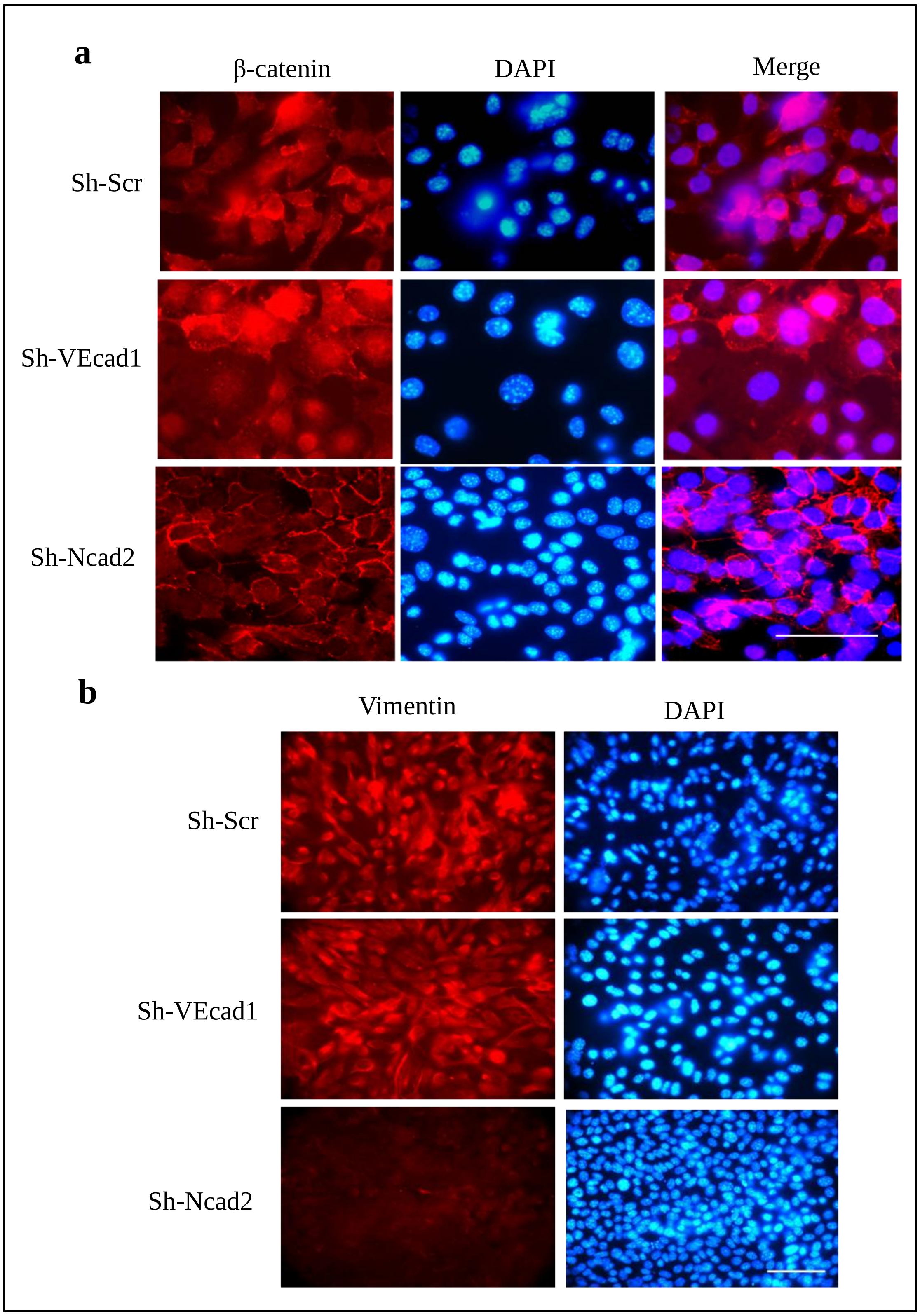

Additional file 6: (a) Immunofluorescence staining of β-catenin in control cell line (Sh-Scr) and Sh-VE-cadherin cell lines (Sh-VEcad1 and Sh-VEcad2). *Bar,* 30 µm. (b) Immunofluorescence staining of vimentin in Sh-Scr, Sh-VEcad1 and Sh-Ncad2 cell lines by immunofluorescence microscopy. *Bar,* 60 µm. Nuclear staining with DAPI is also shown.
