# Supplementary material for: Interplay between neural-cadherin and vascular endothelial-cadherin in breast cancer progression
Source: Breast Cancer Res. 2012 Dec 6;14(6):R154. doi: 10.1186/bcr3367 (PMC4053141; doi:10.1186/bcr3367)
Supplement: Additional file 7 — Immunofluorescence staining of neural (N)-cadherin and vascular endothelial (VE)-cadherin on tumor sections. [file bcr3367-S7.PDF]

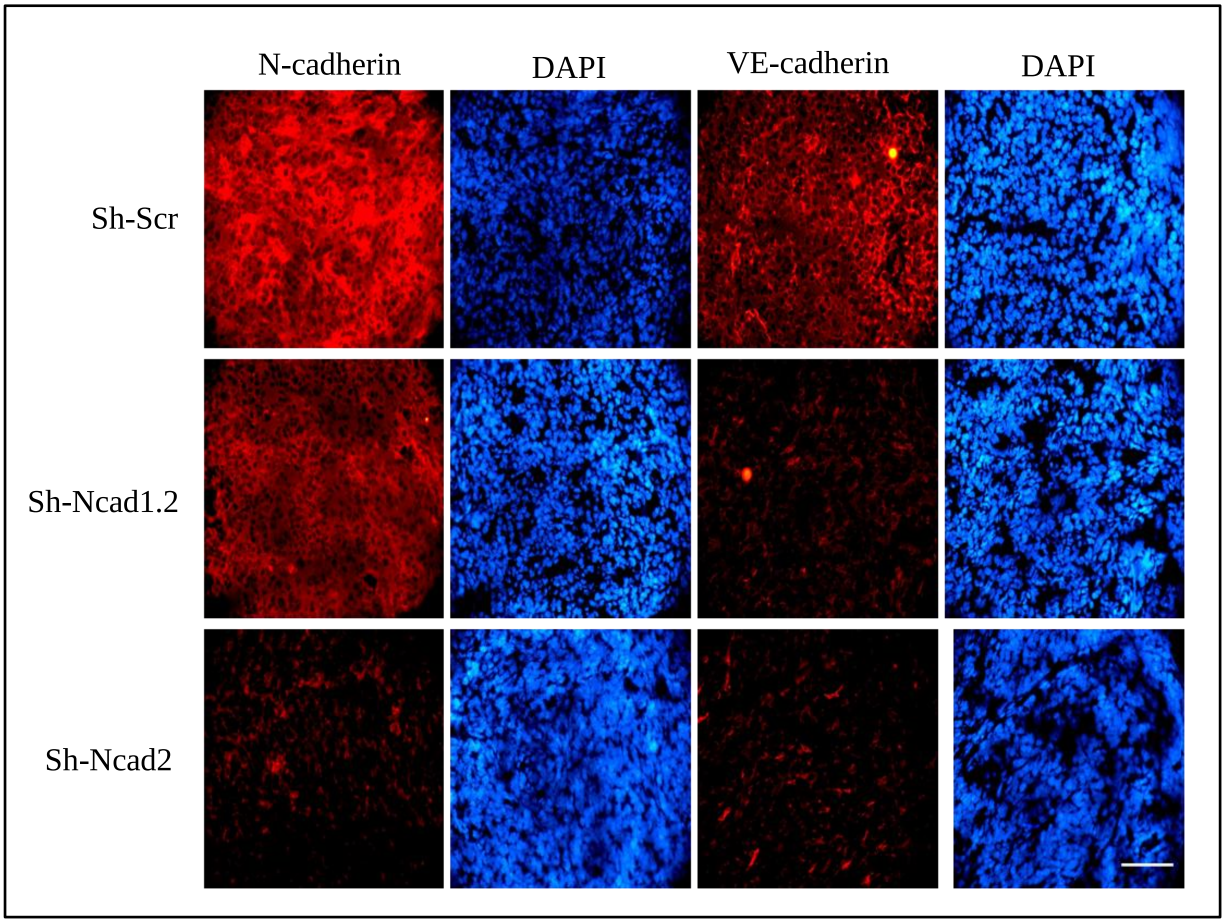


**Additional file 7**: Immunofluorescence staining for N-cadherin and VE-cadherin on tumor sections. *Bar,* 100 µm. Nuclear staining with DAPI is also shown.
