# Supplementary material for: Interplay between neural-cadherin and vascular endothelial-cadherin in breast cancer progression
Source: Breast Cancer Res. 2012 Dec 6;14(6):R154. doi: 10.1186/bcr3367 (PMC4053141; doi:10.1186/bcr3367)
Supplement: Additional file 8 — Morphology and proliferation analysis of vascular endothelial (VE)-cadherin-overexpressing Sh-Ncad2 cells. [file bcr3367-S8.PDF]

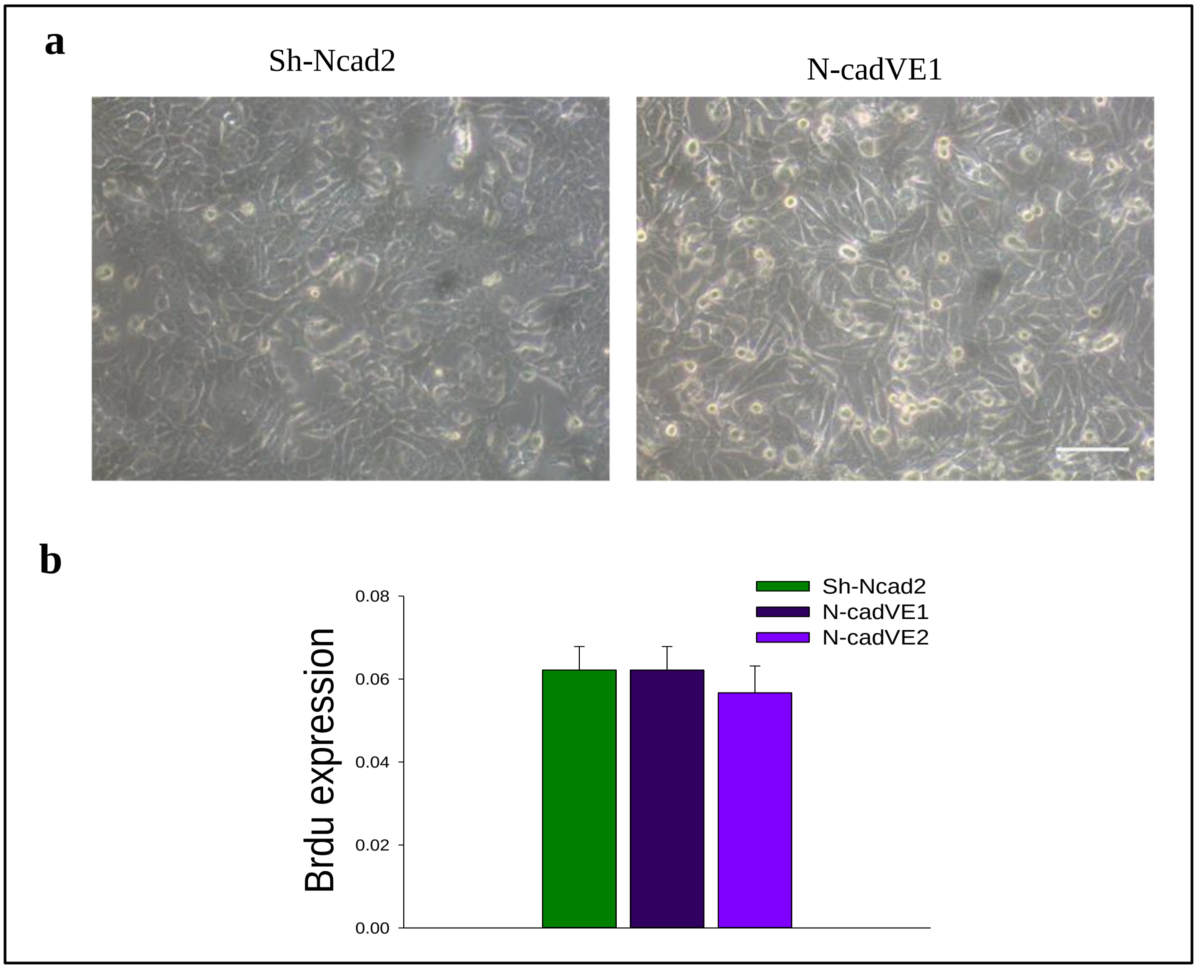

Additional file 8: (a) Phase contrast microscopy of control cell lines (Sh-Ncad2) and Sh-Ncad2 cell line transduced with the VE-cadherin cDNA carrying virus (N-cadVE1). *Bar,* 10 µm (b) 24 hours after culturing equal numbers of cells, cell proliferation was quantified by measuring BrdU incorporation (n=6).
